# Polyethylene Glycol_6000_/carbon Nanodots as Fluorescent Bioimaging Agents

**DOI:** 10.3390/nano10040677

**Published:** 2020-04-03

**Authors:** Chun-Chieh Fu, Chun-Yung Wu, Chih-Ching Chien, Tai-Hao Hsu, Shih-Fu Ou, Shyi-Tien Chen, Chien-Hui Wu, Chien-Te Hsieh, Ruey-Shin Juang, Yi-Huang Hsueh

**Affiliations:** 1 Department of Chemical and Materials Engineering, Chang Gung University, Guishan, Taoyuan 33302, Taiwan; charles07172003@gmail.com; 2 Graduate School of Biotechnology and Bioengineering, Yuan Ze University, Taoyuan 32003, Taiwan; mcyw55@gmail.com (C.-Y.W.); ccchien@saturn.yzu.edu.tw (C.-C.C.); 3 Department of Food Science and Biotechnology, Da-Yeh University, Changhua 51591, Taiwan; th4420@gmail.com; 4 Department of Mold and Die Engineering, National Kaohsiung University of Science and Technology, Kaohsiung 80778, Taiwan; m9203510@nkust.edu.tw; 5 Department of Safety, Health and Environmental Engineering, National Kaohsiung University of Science and Technology, Kaohsiung 81164, Taiwan; shyitien@nkust.edu.tw; 6 Department of SeaFood Science, National Kaohsiung University of Science Kaohsiung 81157, Taiwan; chwu817@gmail.com; 7 Department of Chemical Engineering and Materials Science, Yuan Ze University, Taoyuan 32003, Taiwan; 8 Department of Mechanical, Aerospace and Biomedical Engineering, University of Tennessee, Knoxville, TN 37996, USA; 9 Division of Nephrology, Department of Internal Medicine, Chang Gung Memorial Hospital, Linkou 33375, Taiwan

**Keywords:** carbon quantum dots, nanomaterials, bioimaging, photoluminescence, hydrothermal synthesis

## Abstract

Photoluminescent nanomaterials have immense potential for use in biological systems due to their excellent fluorescent properties and small size. Traditional semiconductor quantum dots are heavy-metal-based and can be highly toxic to living organisms, besides their poor photostability and low biocompatibility. Nano-sized carbon quantum dots and their surface-modified counterparts have shown improved characteristics for imaging purposes. We used 1,3, 6-trinitropyrene (TNP) and polyethylene glycol_6000_ (PEG_6000_) in a hydrothermal method to prepare functional polyethylene glycol_6000_/carbon nanodots (PEG_6000_/CDs) and analyzed their potential in fluorescent staining of different types of bacteria. Our results demonstrated that PEG_6000_/CDs stained the cell pole and septa of gram-positive bacteria *B. Subtilis* and *B. thuringiensis* but not those of gram-negative bacteria. The optimal concentration of these composite nanodots was approximately 100 ppm and exposure times varied across different bacteria. The PEG_6000_/CD composite had better photostability and higher resistance to photobleaching than the commercially available FM4-64. They could emit two wavelengths (red and green) when exposed to two different wavelengths. Therefore, they may be applicable as bioimaging molecules. They can also be used for differentiating different types of bacteria owing to their ability to differentially stain gram-positive and gram-negative bacteria.

## 1. Introduction

Photoluminescent nanomaterials have attracted much attention due to their excellent fluorescent properties and small size. In the past two decades, extensive research on fluorescent semiconductor quantum dots has expanded their application to biology [1,2,3,4,5,6,7,8,9]. However, traditional semiconductor quantum dots (such as PbS and CdTe) usually contain heavy metal ions that are highly toxic to living organisms [10,11,12]. Moreover, low biocompatibility and poor photostability is a hindrance in their development for use in biological systems [5,13,14]. Therefore, there is an imminent need to develop non-metallic quantum dots with better photostability and lower cytotoxicity for use in clinical sciences.

Carbon quantum dots (CQDs), also known as carbon nanodots (CDs), are a lucrative option in the field of sensing, photocatalysis and optoelectronics due to their better fluorescent properties including high quantum yield (*η*), photostability and excitation-related emissions [15,16]. In addition, other advantages of CQDs, including water dispersibility, biocompatibility, lower toxicity, small size, amenable to modifications and low processing cost make them potential material for biomedical applications [5,13,14]. Due to their similar size and photoelectrochemical properties, research on quantum dots has mostly focused on graphene quantum dots, CDs and polymer dots. However, they differ in their internal structure and surface chemical groups, including oxygen-containing groups [17]. For these materials to emit fluorescent light, their sizes and surface chemical groups must be carefully adjusted. The CQDs not only inherit the excellent optical characteristics of traditional semiconductor quantum dots but also make up for the shortcomings of traditional materials in terms of cytotoxicity, environmental and biological hazards; moreover, they can be imparted multi-functionality when conjugated with other nanoparticles. As a result, they have the potential in biomedical applications, such as drug delivery, photosensitizers and carriers for therapeutic and antimicrobial molecules. Significantly, when compared with heavy metal-based semiconductor quantum dots, CQDs show better chemical and optical stability, excellent water dispersion, better biocompatibility and lower cytotoxicity, thereby making them optimal nanomaterial for developing effective nanoprobes in the field of biological imaging.

Tremendous efforts have been made to develop methods for the preparation of CQDs with controllable particle size, high production yield and adjustable surface functionalization [18,19]. By using the three isomers of phenylenediamine, Jiang et al. developed a simple hydrothermal method to synthesize red, green and blue light-emitting CQDs [20]. The absorption spectrum of the CQDs at Ultraviolet- and visible- range showed similar patterns. Also, all three CQD materials underwent redshifts during the absorption transition, indicating that the electronic bandgap of the CQD was smaller than its corresponding precursor. Photoluminescence (PL) is one of the most attractive features of CQD that makes them lucrative for practical applications [21,22]. CQD photoluminescence depends on the emission wavelength and their nanosize with different emission wells on their surfaces [23]. The PL emission changes due to particle size can be reflected in a broad excitation-dependent PL emission spectrum [24]. By studying the emission behavior of CQD at various concentrations at 470 nm, Zhang et al. [25] reported that an increase in the concentration of the CQD solution led to an initial increase followed by a decrease in its PL intensity.

Previous studies had shown that CQDs synthesized by the decomposition of citric acid monohydrate and diethylene glycol bis (3-aminopropyl) have application in fluorescence imaging, with very low cytotoxicity in human Caco-2 cell line and tissues from Kunming mouse liver and kidney [26]. DNA-CQDs synthesized using bacterial DNA could enter the human kidney cell line HEK293 and stain yeast and *E. coli* [27]. Compared to other diagnostic methods, quantum dot nanosized probes are faster, cost-effective and more biocompatible, thereby becoming suitable for in vivo labeling [28]. Moreover, based on their redox potential, they can distinguish cancer cells from normal cells [29,30]. Available literature collectively and strongly suggests quantum dots to be potent as sensitive, efficient and cost-effective diagnostic or imaging nanoprobes [31,32].

So far, multiple methods have been reported for synthesizing CQDs that have resulted in dots with biological properties in bacterial cells [33,34,35,36,37]. However, in most of the studies, the location of these CQDs remained obscure. Hydrothermal method was frequently employed to synthesize CQDs; however, there are few reports focusing on one-pot hydrothermal synthesis of core-shell polyethylene glycol _6000_/carbon nanodots (PEG_6000_/CDs). Herein the polyethylene glycol skin layer would induce a red-shift response due to an increase in particle size of PEG_6000_/CDs. However, the skin layer also acts as a protection layer for an improved biocompatibility, thus leading to an enhanced photo-stability (i.e., a prolonged lifetime). Accordingly, the aim of the present study was to obtain photostable nanodots using a hydrothermal method and to evaluate their potential in biological staining and imaging. This study also highlights the synthesis of PEG_6000_/CDs as differently functionalized carbon nanomaterials using a simple one-step hydrothermal method and demonstrates that their photo-properties make them suitable for application in biological imaging.

*E. coli* K12 has been labelled using a simple drying and heating method to synthesize mannose-modified fluorescent carbon quantum dots (Man-CQDs) from solid amine citrate and mannose [34]. Staining was viewed at excitation and emission wavelengths of 365 and 450 nm, respectively. Bacteria were quantified down to 450 Colony-forming Units (CFU); however, single cells could not be distinguished. Bhamore et al. modified carbon dots from Manilkara heteropoly fruits using sulfuric acid and phosphoric acid to generate blue-, green- and yellow-emitting dots (excitation wavelengths of 350, 420 and 440 nm, respectively and emission wavelengths of 443, 515 and 565 nm, respectively) [35]. These three carbon nanodots could be used for staining cells, *E. coli* and mold. However, cell aggregation hindered the imaging and made the single cells and cell structures unclear. Baig et al. developed an inexpensive chicken protein-based method that could generate carbon dots via a one-step heating reaction [37]. These dots could be used as green markers of *E. coli* and red markers of *Staphylococcus aureus* (emission wavelengths of 450–490 nm and 510–560 nm, respectively) but only on intact cells. Lu et al. used hydrothermal reactions to synthesize nitrogen and phosphorus co-doped carbon dots (NPCD) whose negative charge could be adjusted based on the precursor [36]. By selectively staining the negatively charged cell walls of dead bacteria, NPCD expanded the application of CQDs to bacterial viability assessment in whole bacteria cells. These methods established the utility of CQDs in bio-imaging of bacteria but the question still remained as to whether fluorescent nanocarbon dots could be used for staining specific bacterial cell parts. 

We synthesized polyethylene glycol_6000_/carbon nanodots (PEG_6000_/CDs) as differently functionalized carbon nanomaterials using a simple one-step hydrothermal method, demonstrating that their photo properties were suitable for staining bacterial cell membranes in both Gram-positive and Gram-negative bacteria, as well as cell septa and poles in Gram-positive bacteria.

## 2. Materials and Methods 

### 2.1. Bacterial Strains and Growth Conditions 

*Bacillus subtilis* NCIB3610, *Bacillus thuringiensis* cry 407, *Escherichia coli* K12 and *Pseudomonas aeruginosa* PAO1 were grown and maintained at 37 °C; *E. coli* K12 and *P. aeruginosa* PAO1 were maintained at 30 °C in Luria-Bertani (LB; 10 g tryptone, 5 g yeast extract and 5 g NaCl per liter) broth or on LB plates containing 1.5% Bacto agar [38,39]. For PEG_6000_/CDs and FM4-64 dye staining, bacteria colonies were grown onto LB plates and individual clones were grown in LB medium (180 rpm, 37 °C or 30 °C) until OD_600_ reached approximately 0.5–0.6.

### 2.2. One-Pot Hydrothermal Synthesis of PEG_6000_/CD Composites 

A hydrothermal method was employed to synthesize PEG_6000_/CD composites [40]. Briefly, 2 g of pyrene was added to 240 mL concentrated nitric acid (16 N) and stirred at 200 rpm for 18 h at 80 °C [41]. Here the operating pressure was estimated to be 32 atm. Thereafter, 1 L distilled water was added and the solution was stirred at 150 rpm for another hour. The solution was then subjected to suction filtration and 1,3, 6-trinitropyrene (TNP) was obtained therefrom. Subsequently, 2 g of TNP was added to 2 g of PEG (molecular weight: 6,000) and 50 mL dimethylformamide (DMF) and sonicated in a polytetrafluoroethylene (PTFE) tank for 1 h. The solution allowed to react hydrothermally for 12 h at 180 °C in a stainless-steel autoclave, thereby producing PEG_6000_/CD composites. The material was purified using centrifuged at high speed to separate the big size particle and take supernatant. The PEG_6000_/CD samples were centrifuged at three times at 13,000 rpm, for 5 min each times and take supernatant and do centrifuged again and finally we filtered supernatant using a 0.22 μm membrane filter. The production yield of PEG_6000_/CD composites was approximately 56 wt.%. 

### 2.3. PEG_6000_/CDs Characterization

A high-resolution transmission electron microscope (HR-TEM, FEI Talos F200S, Thermo Fisher Scientific, Waltham, MA, USA) operating at 200 kV was employed to capture the micrographs of PEG_6000_/CD samples. X-ray photoelectron spectroscopy (XPS, Fison VG ESCA210, West Sussex, England) equipped with Mg-K*α* radiation emitter, was used to characterize the chemical composition of the samples. The C 1s, N 1s and O 1s spectra were deconvoluted utilizing a non-linear least square fitting algorithm with a symmetric Gaussian function. Surface composition of the samples was further characterized by applying an appropriate sensitivity factor. Ultraviolet-visible (UV-vis) spectra of PEG_6000_/CD suspensions were recorded using Jasco FP-8200 spectrometer, in which the wavelength scan rate was set at 60 nm/min. The PEG_6000_/CD suspension was prepared by dispersing a solid (100 mg) into 1000 mL solvent, containing distilled water and DMF (50/50 in *v*/*v*). The PL emission spectra of each suspension were carried out by a fluorescence spectrometer (F-7000 FLS920P, Hitachi, Tokyo, Japan) at 360, 490 and 557 nm. The *η* value of the sample was measured, referred to Coumarin (C_9_H_6_O_2_, molecular weight: 146) reference (*η*: 73% at 360 nm excitation). The *η* value of each CD sample was determined by the equation [42,43]:

*η* = *η*_r_ × [(PL area/OD)_s_/(PL area/OD)_r_] × *Φ*_s_^2^/*Φ*_r_^2^

where the s and r subscripts represent the CD sample and the reference (i.e., Coumarin), respectively. Herein *Φ* represents the reflective index of solvent and PL area and OD are the PL spectral area and absorbance value, respectively. All experimental data are representative of results derived from three separate experiments.

### 2.4. Carbon Quantum Dots Staining 

Three mL of bacterial cells growing at log phase (OD_600_ ~0.5) were centrifuged at 14,000 rpm for 3 min. The pellets were washed with 1× TBS buffer and resuspended in 50 μL PEG_6000_/CD at various concentrations (12.5, 25, 50 and 100 ppm) and incubated at room temperature for 1 min. The cultures were centrifuged at 14,000 rpm for 3 min and pellets were washed twice with 1× TBS buffer. A total of 10 μL of the stained cells were placed on a slide covered with a poly-L-Lysine coated coverslip. The images were captured using a fluorescence microscope (Axio Lab A1, Carl Zeiss, Göttingen, Germany) fitted with a Zeiss Axiocam 503 color camera (Carl Zeiss, Göttingen, Germany) and an attached VIS-LED lamp (Carl Zeiss, Göttingen, Germany). All experimental data are representative of results derived from three separate experiments.

### 2.5. Fluorescence Microscopic Analysis of Bacterial Cells 

The cells were analyzed for red tetramethylrhodamine (TRITC) filter at 527.5–552.5 nm excitation wavelength and 590–650 nm emission wavelength; 0.45 seconds exposure time) and green fluorescence fluorescein isothiocyanate (FITC) filter at 465–495 nm excitation wavelength and 515–555 nm emission wavelength; 1.6 seconds exposure time). The phase-contrast images were acquired in the BF filter under auto exposure time. For FM4-64 imaging, the pellets were resuspended in 50 μL FM4-64 (50 μg/mL) and incubated for 3 min in dark at room temperature. A total of 10 μL of the stained cells were placed on a slide covered with a poly-L-Lysine coated coverslip. The images were acquired using a fluorescence microscope. For quenching the experiments, the cells were photo-irradiated through a 557 nm filter coupled for 3 and 30 seconds, respectively, followed by re-imaging the cells using the TRITC filter as described earlier. All experimental data are representative of results derived from three separate experiments.

## 3. Results and Discussion

### 3.1. Structural and PL Analysis of PEG_6000_/CDs

**Figure 1:**
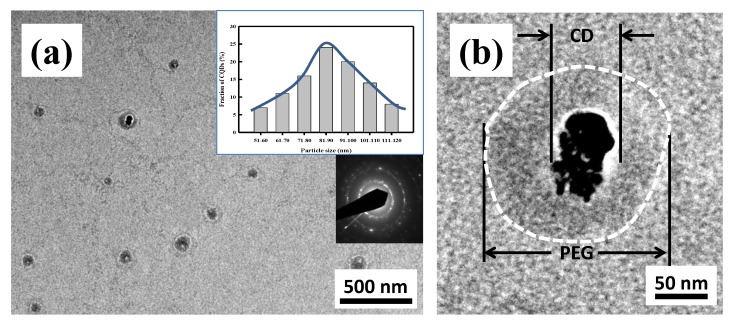
High-resolution transmission electron microscope (HR-TEM) micrographs of polyethylene glycol_6000_/carbon nanodots (PEG_6000_/CDs) particles with low (**a**) and (**b**) high magnification. The inset shows the particle size distribution and the selected-area diffraction pattern on the PEG_6000_/CD particles.

The PEG_6000_/CDs were prepared by the hydrothermal method and their crystal structures were subjected to transmission electron microscopy (TEM) and X-ray diffraction (XRD) analyses. Results from the TEM analysis showed that PEG_6000_/CDs formed a structure with carbon nanodots at the center and polyethylene glycol coating outside (Figure 1a). We observed that the carbon nanodots have an average particle size of 30 nm, based on HR-TEM micrograph. After the deposition of PEG skin layer, the PEG_6000_/CDs tend to increase in their particle sizes. High-resolution TEM and dynamic light scattering (DLS) particle size analyzers were used to count 300 dots from 3 different spots and the data was used to analyze the average PEG_6000_/CDs (Figure 1b). The particle size distribution of PEG_6000_/CDs was also given in the inset of Figure 1a, showing a quasi-Gaussian distribution, determined from the DLS analysis. This result reveals that the average size of these dots is approximately 87 nm. The inset also displays a selected-area diffraction pattern of individual PEG_6000_/CD particle, indicating the bright diffraction spots along with diffraction rings. The presence of such bright rings suggests that the CDs consisted of nano-crystallites [42].

**Figure 2:**
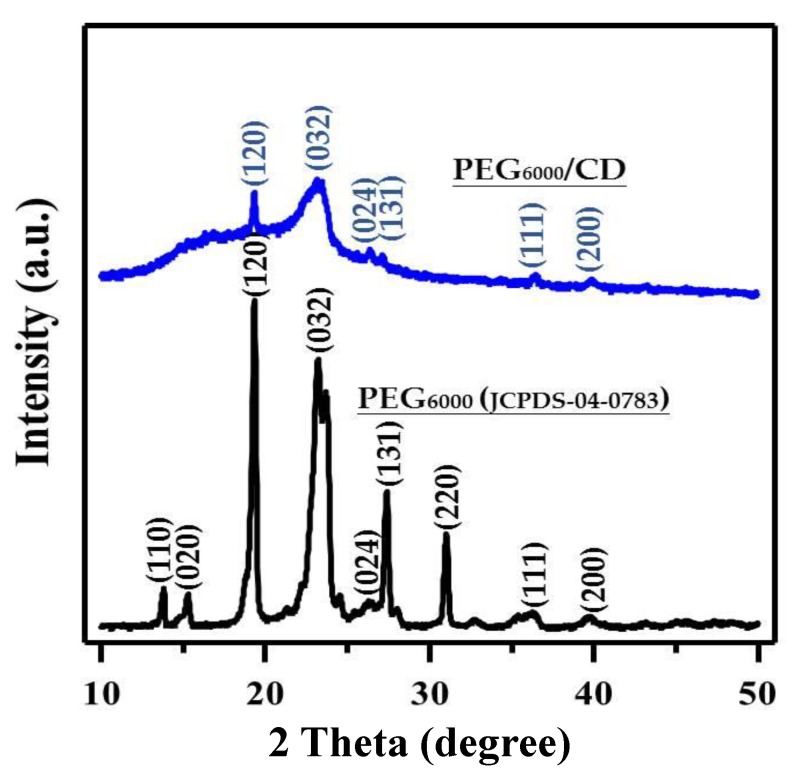
X-ray diffraction (XRD) pattern of PEG_6000_/CD composite. Here the standard XRD pattern of polyethylene glycol_6000_ (PEG_6000_) is given for comparison (JCPDS-04-0783).

Next, the PEG_6000_/CDs were subjected to XRD analysis and compared with the standard spectra of PEG JCPDS04-0783. As can be seen in Figure 2, the scattering angle (2*θ*) and the crystal plane of PEG_6000_ were 13.8° (110), 14.8° (020), 19.3° (120), 23.2° (032), 27.0° (024), 27.4° (131), 31.0° (220), 36.4° (111) and 43.1° (200). However, the PEG_6000_/CD composites lacked three peaks at 13. 8° (110), 14.8° (020) and 31.0° (220). In addition, the amorphous peak of the carbon nanodots was located at 23.2° (032), proving that PEG_6000_/CDs did not crystallize well during the 180°C hydrothermal preparation process; however, they exhibited the characteristic peaks of PEG_6000_, thereby showing that the PEG_6000_ and CDs bonded to form a composite material. The weak lump occurs at *ca*. 23.2°, assigned to the (002) crystal plane of graphite-like structure. According to the Bragg’s Law [44,45], the inter-planar distance (*d*_(002)_) is calculated to be 0.383 nm, which is larger than that of ideal interlayer distance of highly oriented graphite (*d*_(002)_: 0.335 nm).

**Figure 3:**
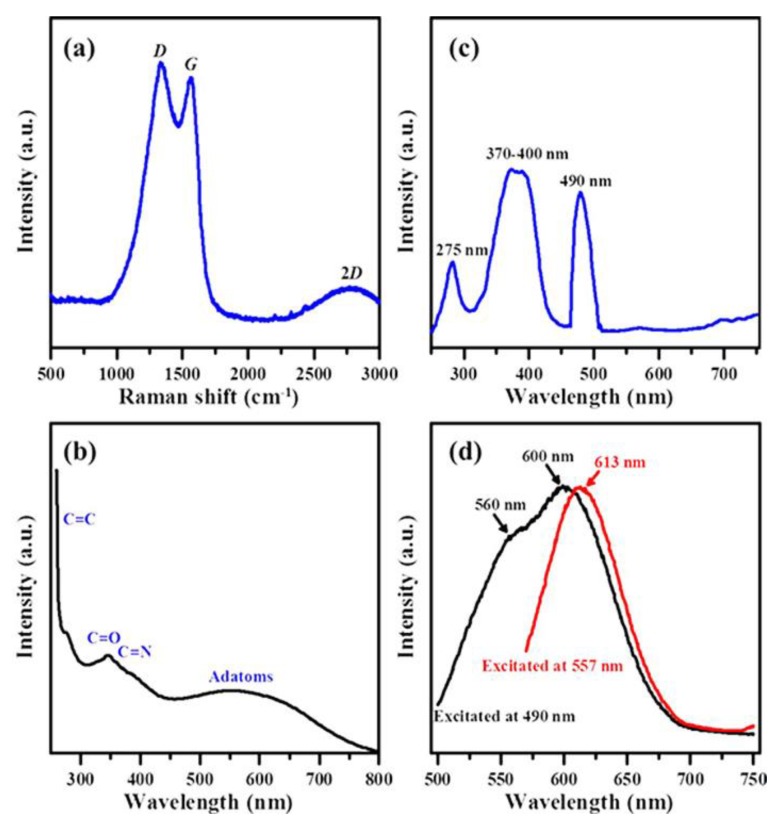
(**a**) Raman spectra of PEG_6000_/CD sample measured with 514 nm argon laser at 10 mW power, (**b**) UV-vis absorbance spectrum and (**c**) Photoluminescence (PL) excitation and (**d**) PL emission spectra of PEG_6000_/ CDs excited at 490 and 557 nm.

The results of the Raman spectrum analysis showed that the peaks of the CQDs were located at 1359 cm^−1^ (*D*) and 1598 cm^−1^ (*G*), while the special 2*D* peak was located at 2700 cm^−1^, as shown in Figure 3a. It is generally recognized that the Raman band observed at *ca*. 1580 cm^−^^1^ is ascribed to a single crystallite of graphite (*G* band), whereas the *D* band at *ca*. 1350 cm^−^^1^ is commonly attributed to amorphous carbon or deformation vibrations of a hexagonal ring [46]. The *I_D_*/*I_G_* ratio was *ca*. 1.05, revealing that the PEG_6000_/CDs composite obtained using the hydrothermal route have a mixed crystallinity with highly amorphous carbons. The UV-vis absorbance spectrum show a typical π–π* transition absorption peak due to the aromatic sp^2^ domains (C=C) around 270 nm, an *n*–π* transition absorption peak due to C=O/C=N bonding around 340 nm, a band-gap transition absorption band due to surface molecular center or absorption edge induced by *n*–π* transitions of nonbonding electrons of adatoms around 500–600 nm [47,48,49] and a long tail extending into the visible range in Figure 3b. 

The analysis of the PL spectrum shows the best excitation energy (or wavelength) and the strongest emission range for a phosphor. As viewed, the spectrum reveals three peaks at *ca*. 275 nm, 370–400 nm and 490 nm (Figure 3c). Among these, the peak at 490 nm was quite different from the general CQDs and it could be caused due to the presence of PEG_6000_. The excitation light spectrum analysis of PEG_6000_/CDs composite materials at 490 nm and 557 nm is shown in Figure 3d. It is worth mentioning that the other two excitation sources (i.e., 490 and 557 nm) were used for the bioimaging measurement. This is because the wavelengths are frequently used in bio-fluorescence microscopy. Our results showed that for excitation at 490 and 570 nm, the corresponding emission peaks were obtained at about 560 nm, 600 nm (approximately red) and 613 nm (biased and nearly green). These results showed that the TRITC filter (527.5–552.5 nm excitation wavelength and 590–650 nm emission wavelength) and the FITC filter (465–495 nm excitation wavelength and 515–555 nm emission) could be used to detect the presence of PEG_6000_/CDs. The *η* value of 6.98% is achieved by PEG_6000_/CDs, prepared from the hydrothermal synthesis of chemical precursor.

### 3.2. Surface Functionalization and Inter-Band Gap Model of PEG_6000_/CDs

**Figure 4:**
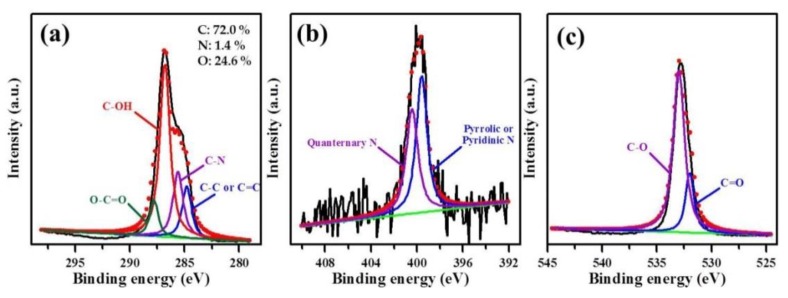
XPS spectra of PEG_6000_/CD sample: (**a**) O 1s peak, (**b**) N 1s peak and (**c**) O 1s peak, deconvoluted by a multiple Gaussian function.

We used X-ray photoelectron spectroscopy (XPS) to determine the surface chemical composition of the prepared PEG_6000_/CDs and the results are shown in Figure 4. Our results showed that in addition to the characteristic peak at 284.5 eV indicating C-C or C=C bond, other peaks characteristic of C–N, C–OH and O=C–O bonds appeared at 285.2 eV, 287.5 eV and 288.5 eV, respectively (Figure 4a). The characteristic peaks at 399.6 eV and 400.6 eV indicated the presence of pyrrolic/pyridinic N and quaternary amine (quaternary N), respectively (Figure 4b). Further, the high-intensity peaks at approximately 532.5 eV and 531.8 eV indicated C–O and C=O, respectively (Figure 4c). 

The molar ratios of C–C/C=C, C–N, C–OH and O=C–O were 14.8%, 20.7%, 55.0% and 9.5%, respectively (Table 1). Among the other functional groups present, the content of C–N group was as high as 20.7%, indicating *N* dopants in the carbon ring structure. The ratio of quaternary nitrogen atoms in pyrrolic to pyridinic species was 54.3%:45.7%. These findings indicated that the formation of TNP could be implemented in the CDs composed of carbon rings and N dopants in the subsequent processes.

**Figure 5:**
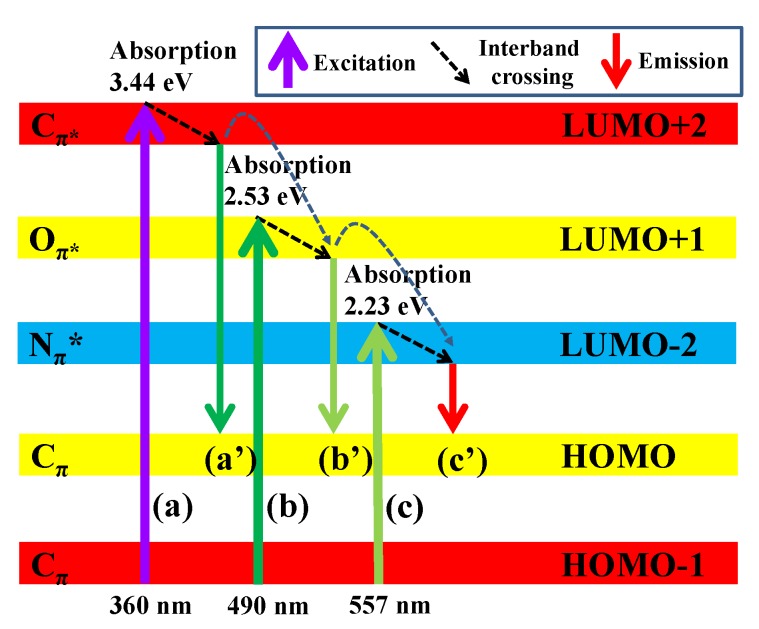
Inter-band gap structure model of PEG_6000_/CDs under different light illuminations.

Accordingly, it is inferred from the above observations that the N and O doping alter the energy levels of PEG_6000_/CDs composite. Herein we proposed a schematic diagram of different electronic transitions on PEG_6000_/CDs, as shown in Figure 5, implying that the inter-band gap structure model of PEG_6000_/CDs cannot obey the Kasha’s rule. The individual transitions are indicated by arrows in the energy band diagram, that is, a multiple chromophoric band-gap structure [43,49,50,51,52], which is related to photon rejection from the highest occupied molecular orbital (HOMO)-lowest unoccupied molecular orbital (LUMO) gap. The decoration of O and doping of N atoms are prone to create additional “defect sites,” thereby introducing additional energy levels and effectively creating new electron transition pathways in the inter-band structure of CDs [49,53]. Under UV-light irradiation, the absorption of UV photons by the localized π electron in double bonds (mainly C=C) produces an electron-hole pair (exciton) after electron transition, that is, path (a). The exciton may emit UV light (path (a’)) through radiative recombination after vibration relaxation. The excited electron may undergo inter-band transition from a higher conduction band to a lower conduction band (e.g., path (b) and (c)), subsequently emitting visible light (paths (b’) and (c’)) by radiative recombination [54]. This inter-band structure also covers the reason why PEG_6000_/CD sample emit an asymmetric PL emission spectrum (similar to a twin PL emission) under 490-nm irradiation (path: (b)-(b’) and (c)-(c’)), whereas a quasi-symmetric PL emission can be observed at 557-nm illumination (path: (c)-(c’)). As compared to the *η* value (= 6.98% at 360 nm), we observed that both *η* values at 490 and 557 nm decrease to 1.70 and 1.08%, respectively. On the basis of the deduction, the decreasing trend in *η* value reveals that the photon pathway from high energy adsorbing site to low energy emissive site is attributed to the excitation light intensity and the presence of N and O functional groups on PEG_6000_/CD sample.

### 3.3. Fluorescent Bioimaging Using PEG_6000_/CD composite

**Figure 6:**
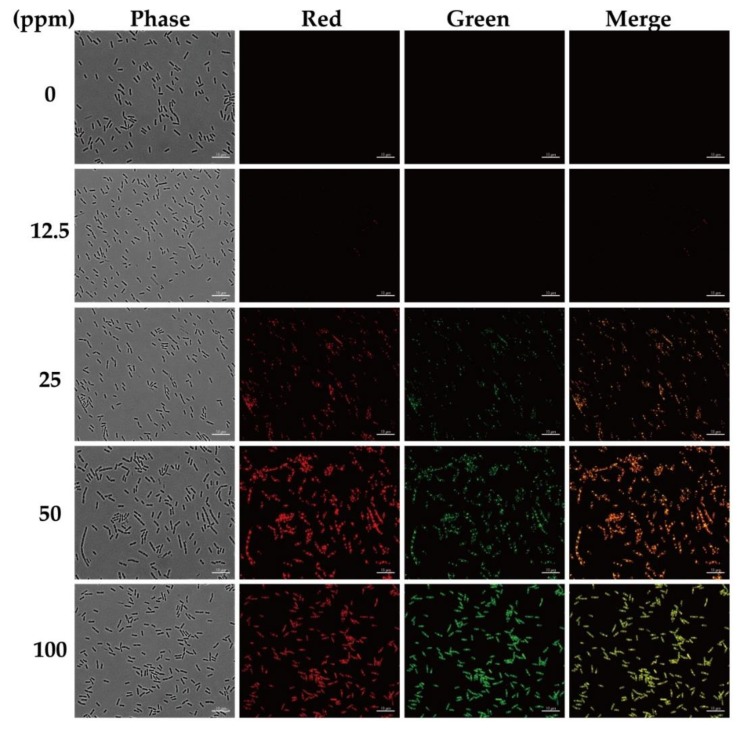
Fluorescence microscopy images of *B. subtilis* stained with PEG_6000_/CDs. Cells were stained with different concentrations of PEG_6000_/CDs (0, 12.5, 25, 50 and 100 ppm). Control cells were assayed similarly without adding PEG_6000_/CDs. TRITC filter detected red color, while FITC filter detected green color. Scale bar represents 10 μm.

Next, we used different concentrations of PEG_6000_/CDs composite to stain gram-positive bacterium *B. subtilis* at room temperature and analyzed the cells using a Zeiss fluorescence microscope fitted with a Zeiss Axiocam 503 color camera and Vis-LED light. The staining results using various concentrations (0–100 ppm) are shown in Figure 6.

**Figure 7:**
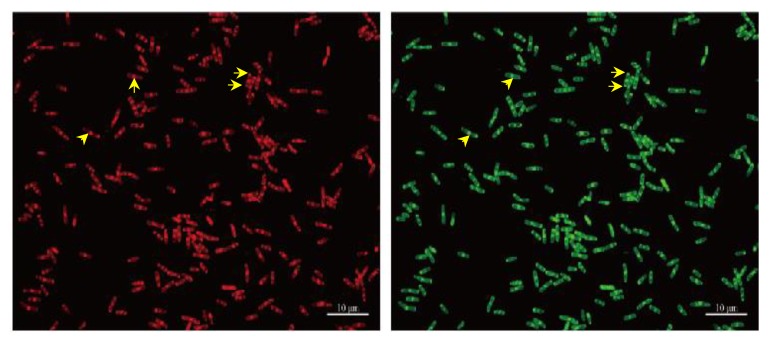
Fluorescence microscopy images of *B. subtilis* stained with PEG_6000_/CDs. Cells were stained with the concentrations of PEG_6000_/CD at 100 ppm. TRITC filter detected red color, while FITC filter detected green color. Scale bar represents 10 μm. Yellow arrows point to cell septa and cell poles.

The composite stained the cells at all the tested concentrations but the best staining was observed at 100 ppm. Significantly, similar results were observed with TRITC and FITC filters. An enlarged view of PEG_6000_/CDs stained *B. subtilis* is shown in Figure 7. The yellow arrows indicate that in addition to staining cell membranes, PEG_6000_/CDs composites also stained the septum and cell poles. 

The XPS of PEG_6000_/CDs showed that C–N, C–OH and O=C–O corresponded to 20.7%, 55.0% and 9.5%, respectively (Table 1); these functional groups may interact with the proteins in the septum and pole via hydrogen bonding. For example, O=C–O can interact with NH_2_ and C–N can interact with COOH in polypeptides. These interactions may explain the PEG_6000_/CDs staining and accumulation in the septa and poles. In addition, when compared to Gram-positive bacteria, Gram-negative bacteria have two lipid bilayer membranes (inner and outer membranes) that may make it more difficult for PEG_6000_/CDs to enter them. Hence, staining of the septa and pole may have been easier in *B. subtilis* since it only had one thin membrane.

**Figure 8:**
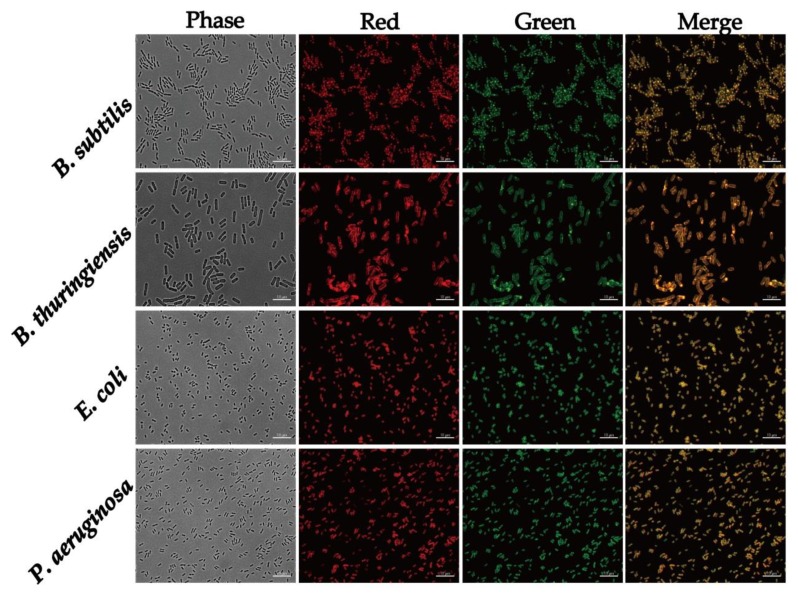
Fluorescence microscopy images of *B. subtilis, B. thuringiensis, E. coli and P. aeruginosa* cells stained with PEG_6000_/CD at 100 ppm. TRITC filter detected red color and FITC filter detected green color. Scale bar represents 10 μm.

Next, we tested the composite to stain different bacteria such as *B. thuringiensis* 407 (gram-positive), *E. coli* K12 (gram-negative) and *P. aeruginosa* PAO1 (gram-negative). Our results showed that the PEG_6000_/CD composite stained all the bacterial types (Figure 8). The composites stained the whole cell and membranes of *P. aeruginosa* PAO1, while membranes were stained in all three bacteria. Interestingly, the composite stained the membrane and septa in *B. thuringiensis* 407 and *B. subtilis* 3610. This differential staining might be explained by the different membrane composition of the gram-positive and gram-negative bacteria. 

**Figure 9:**
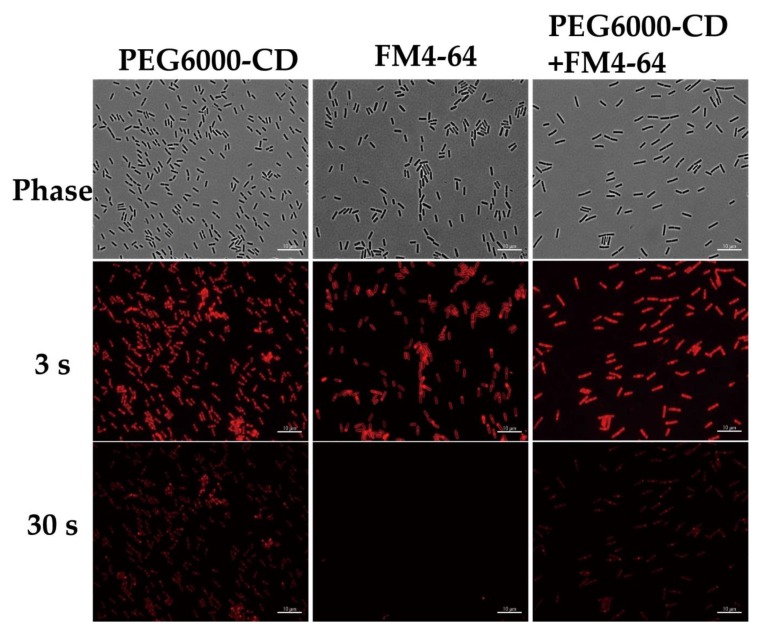
Fluorescence microscopy images of *B. subtilis* stained with PEG_6000_/CDs and FM4-64 membrane dye and excited with t 490 nm wavelength for two different durations (3 s and 30 s) before imaging. Cells were stained with PEG_6000_/CDs at 100 ppm. Control cells were assayed similarly without adding PEG_6000_/CDs. TRITC filter detected red color. Scale bar represents 10 μm.

Finally, the fluorescence quenching experiments were performed to analyze the photostability of the FM4-64 and PEG_6000_/CD in *B. subtilis* cells using a laser (557 nm, 3 and 30 s), followed by exposure and analysis. Our results showed that while the commercially available FM4-64 dye was bleached after 30 s of irradiation, cells retained the PEG_6000_/CD staining indicating that PEG_6000_/CD had better photostability and therefore have potential role in cellular imagining (Figure 9). FM4-64 only has two fluorescent carbon aromatic rings but PEG_6000_/CD has 10 to 20 aromatic condensed rings response to fluorescent light emission. This might explain why FM-464 is easier to quenching than PEG_6000_/CD.

## 4. Conclusions

We have developed a simple hydrothermal method to synthesize PEG_6000_/CD composites and have evaluated their function as bio-labels using four different bacteria. Our results indicated that the fluorescence properties of PEG_6000_/CDs did not differ significantly across the tested bacteria. Further, PEG_6000_/CDs stained the cell pole and septum of gram-positive bacteria only. Further, our results showed that optimal staining was achieved at approximately 100 ppm. Compared to the commercially available membrane dye FM4-64, PEG_6000_/CD composites exhibited better photostability and higher resistance to photobleaching. Unlike FM4-64, PEG_6000_/CDs could emit two different colors (red and green) when exposed to two different wavelengths. Collectively, our results showed the possibility of synthesizing PEG_6000_/CDs as differently functionalized carbon nanomaterials using a simple one-step hydrothermal method and demonstrated their photo-properties that make them suitable for application in biological imaging. 

## Figures and Tables

**Table 1:** XPS analysis showing different percentages of bonding groups in PEG_6000_/CD sample.

Synthesized Sample	Element Ratio (mol %)
C 1s peak	
C–C	14.8
C–N	20.7
C–OH	55.0
O–C=O	9.5
N 1s peak	
Quaternary N	54.3
Pyrrolic/Pyridinic N	45.7
O 1s peak	
C=O	21.6
C–O	78.4
